# BRAF-induced *EHF* Expression Affects *TERT* in Aggressive Papillary Thyroid Cancer

**DOI:** 10.1210/clinem/dgae589

**Published:** 2024-08-26

**Authors:** Yiyi Xu, Jiwei Gao, Na Wang, Jan Zedenius, Inga-Lena Nilsson, Weng-Onn Lui, Dawei Xu, C Christofer Juhlin, Catharina Larsson, Ninni Mu

**Affiliations:** Department of Oncology-Pathology, Karolinska Institutet, Stockholm SE-171 64, Sweden; Department of Oncology-Pathology, Karolinska Institutet, Stockholm SE-171 64, Sweden; The Cancer Hospital of the University of Chinese Academy of Sciences, (Zhejiang Cancer Hospital), Hangzhou 310022, China; Department of Medicine-Huddinge, Karolinska Institutet, Stockholm SE-141 83, Sweden; Department of Molecular Medicine and Surgery, Karolinska Institutet, Stockholm SE-171 76, Sweden; Department of Breast, Endocrine Tumors and Sarcoma, Karolinska University Hospital, Stockholm SE-171 64, Sweden; Department of Molecular Medicine and Surgery, Karolinska Institutet, Stockholm SE-171 76, Sweden; Department of Breast, Endocrine Tumors and Sarcoma, Karolinska University Hospital, Stockholm SE-171 64, Sweden; Department of Oncology-Pathology, Karolinska Institutet, Stockholm SE-171 64, Sweden; Department of Medicine-Solna, Karolinska Institutet, Stockholm SE-171 76, Sweden; Department of Oncology-Pathology, Karolinska Institutet, Stockholm SE-171 64, Sweden; Department of Pathology and Cancer Diagnostics, Karolinska University Hospital, Stockholm SE-171 64, Sweden; Department of Oncology-Pathology, Karolinska Institutet, Stockholm SE-171 64, Sweden; Department of Oncology-Pathology, Karolinska Institutet, Stockholm SE-171 64, Sweden; Clinical Genetics, Karolinska University Hospital, Stockholm SE-171 76, Sweden

**Keywords:** EHF, TERT promoter mutation, BRAF^V600E^, thyroid carcinoma, prognosis

## Abstract

**Context:**

*BRAF^V600E^* and *TERT* promoter mutations in papillary thyroid carcinoma (PTC) have a synergistic effect on prognosis. This effect is believed to arise from MAPK activation triggered by BRAF^V600E^, leading to the upregulation of ETS transcription factors that bind to the mutant *TERT* promoter.

**Objectives:**

To explore the role of ETS factors in relation to clinical features, *BRAF^V600E^*, and *TERT* promoter mutations in PTC.

**Design:**

Transcriptomic data for 28 ETS factors were analyzed in the PTC cohort of The Cancer Genome Atlas (n = 399) and subsequently validated in a local cohort (n = 93). In vitro experiments were performed to investigate the regulatory role in relation to *BRAF^V600E^* and *TERT* expression.

**Results:**

The Cancer Genome Atlas identified *ETS1*, *ERG*, *FLI1*, *GABPA*, *EHF*, *ETV6,* and *SPDEF* as differentially expressed genes between stages I + II and III + IV. In both cohorts, *EHF* was consistently associated with adverse clinical features, *BRAF^V600E^* and *TERT* promoter mutation/expression. Notably, in *BRAF^V600E^* mutated PTC, high *EHF* expression was associated with shorter disease-free survival. Cases harboring concurrent *BRAF^V600E^*, *TERT* promoter mutations, and high *EHF* expression exhibited the shortest disease-free survival. In cells harboring concurrent *BRAF^V600E^* and *TERT* promoter mutation, overexpression of EHF significantly increased *TERT* expression, whereas knockdown or pharmacological inhibition of BRAF significantly decreased both *EHF* and *TERT* expression. In addition, chromatin immunoprecipitation and quantitative PCR analysis suggested a potential binding of EHF in *TERT* promoter mutant cells but not in *TERT* promoter wild-type cells.

**Conclusion:**

The ETS transcription factor *EHF* is associated with poor prognosis in PTC. This is potentially mediated by BRAF-induced upregulation of *EHF*, which in turn increases *TERT* expression in *TERT* promoter mutated cells.

Papillary thyroid carcinoma (PTC) is the most common histological subtype of follicular-cell derived thyroid carcinoma (TC), accounting for more than 80% of all TCs, and its incidence is increasing worldwide (1). Although PTC patients generally show an excellent prognosis, a subset of patients with PTC exhibit regional recurrences and/or distant metastasis (2), and rare cases dedifferentiate to poorly differentiated thyroid carcinoma or anaplastic thyroid carcinoma (ATC). Therefore, it remains important to elucidate the underlying molecular events leading to the development and progression of PTC to improve prognostication and develop novel approaches to targeted therapy.

We and others have identified activating promoter mutations of the telomerase reverse transcriptase (*TERT*) gene as a recurrent event in TC with impact on poor clinical outcomes (3-6). In PTC, *TERT* promoter mutations are frequent in older patients and have been associated with shorter survival, aggressive PTC features, and loss of radioiodine uptake (3-7). The 2 hotspot *TERT* promoter mutations creates a novel binding site for ETS transcription factors, resulting in *TERT* transcription and telomerase activation (8, 9). Previously, it was reported that the co-occurrence of *TERT* promoter mutation and *BRAF^V600E^* point mutation have a synergistic effect on poor prognosis in PTC (10, 11). *BRAF^V600E^* is the most common genetic driver event in PTC (12, 13), but its relevance as an independent prognostic factor is controversial (14, 15). It has been suggested that the synergistic effect of concurrent *BRAF^V600E^* and *TERT* promoter mutation is mediated by activation of the MAPK pathway induced by *BRAF^V600E^*, which upregulates ETS transcription factors that binds to the mutant *TERT* promoter, inducing *TERT* expression (16).

The ETS family includes 28 transcription factors that have all been associated with cancer development (17). Both up-regulation and down-regulation of ETS transcription factors have been reported as associated with tumor development or progression depending on the type of cancer (17). Increasing evidence has been accumulating suggesting that ETS factors play an important functional role in TC including PTC (16, 18-21). For example, ETS1 and ETS2 have been implicated in the malignant transformation of TC cells (18), whereas ETV1, ETV4, ETV5, and ELK1 play a role in PTC by regulating *TERT* (16, 19, 20, 22). Also, the dysregulation of some ETS factors, including ETV5, ELF3, and GABPB1, is associated with aggressive clinical features (23-25). However, a comprehensive investigation of their potential clinical significance is still lacking. Therefore, we systematically explored the role of the ETS transcription factors in relation to clinical features in PTC.

## Materials and Methods

### The Cancer Genome Atlas Data set of PTC Cases (PTC_TCGA_)

The Cancer Genome Atlas (TCGA) data sets of PTC_TCGA_, including clinical information, genomic data, and mRNA expression profiles were downloaded in October 2019 from cBioPortal (http://www.cbioportal.org) or GDC Data Portal (https://portal.gdc.cancer.gov). A total of 399 PTC cases were analyzed after exclusion of cases with follicular variant of PTC to avoid inclusion of noninvasive follicular thyroid neoplasms with papillary-like nuclear features (NIFTPs) based on the World Health Organization (WHO) classification (26, 27). Genomic data for *TERT* promoter mutation status were downloaded (13). Gene expression data were obtained for *TERT* and the 28 ETS transcription factors. In addition, for selected ETS factors, expression data were downloaded for 58 samples of adjacent nontumorous thyroid tissue (NT_TCGA_) for comparison between normal thyroid and PTC.

### PTC Patients and Tumor Samples

Fresh frozen tissue from 93 primary PTCs derived from patients operated at the Karolinska University Hospital within the period 1987 to 2005 (PTC_K_) were included. The inclusion and exclusion criteria of patients were previously described in detail (21). All patients were diagnosed in clinical routine according to the 2004 WHO classification of endocrine tumors (28). To avoid inclusion of cases with NIFTP based on the WHO classification (26, 27), we excluded all cases annotated as follicular variant of PTCs from the study. Tumor specimens were kept frozen in −70 °C and reviewed by an endocrine pathologist (C.C.J.) for tumor cell representation before inclusion. Clinical information and follow-up data were collected retrospectively. The median follow-up time for overall and disease-free survival was 14.8 years and 13.5 years, respectively. Analyses of *BRAF^V600E^* and *TERT* promoter mutations and *TERT* mRNA expression have been previously published (21). The study was approved by the Swedish Ethical Review Authority. All patients gave their informed consent before inclusion in the study.

### Cell Lines and Cell Culture

All cell lines and sources are summarized in the Supplementary Table S1 (29). The PTC (MDA-T32, RRID: CVCL_W913 and MDA-T41, RRID: CVCL_W914) and ATC-derived cell lines (hth-104, RRID: CVCL_A427; SW1736, RRID: CVCL_3883; hth-74, RRID: CVCL_6288; and hth-112, RRID: CVCL_A428) were cultured in RPMI-1640 medium (Thermo Fisher Scientific) with 10% fetal bovine serum (Thermo Fisher Scientific) and 1% nonessential amino acids. The melanoma cell line A375 (RRID: CVCL_A375), was cultured in DMEM medium supplemented with 10% fetal bovine serum, whereas SK-MEL-3 (RRID: CVCL_0550) was cultured in McCoy's 5A (modified). *BRAF^V600E^* and *TERT* promoter mutation status of all cell lines were verified by Sanger sequencing and matched to previous publications (Supplementary Table S1) (21, 29-31). Short tandem repeat genotyping was performed for authentication of PTC and melanoma cell lines (Supplementary Table S2) and has been previously published for ATC cell lines (21, 29). All cell lines were grown at 37 °C and 5% CO_2_.

### Differentially Expressed Gene Analysis

To identify differentially expressed ETS transcription factors related to aggressive phenotype, the PTC_TCGA_ cohort was grouped based on disease stage according to the 7th edition of the American Joint Committee on Cancer for analysis. Stage I + II (n = 252) and stage III + IV (n = 142), respectively, were grouped for comparison of expression levels for the 28 ETS transcription factors. Raw read counts for each gene were analyzed using DESeq2. The Benjamini-Hochberg correction was applied to correct for multiple testing and genes with an adjusted *P* value <.05 were considered as significantly differentially expressed genes (DEGs). mRNA expressions are presented as RSEM (RNA-seq by expectation-maximization) or log2(x + 1) transformed RNA-seq by expectation-maximization normalized counts in figures.

### RNA Extraction and Reverse Transcription Quantitative PCR

Total RNA was extracted from cell lines and PTC_K_ tumor samples using mirVana miRNA Isolation Kit (Invitrogen, AM1560) and quantified with NanoDrop ND-100 spectrophotometer (Nano Drop Technologies). Reverse transcription of RNA was performed using High-Capacity cDNA Reverse Transcription Kit (Applied Biosystems). Reverse transcriptase quantitative PCR (RT-qPCR) of PTC_K_ was performed with Taqman Gene expression assays for *EHF* (Hs00171917_m1), *ERG* (Hs01554629_m1), *FLI1* (Hs00956709_m1), *ETV6* (Hs00231101_m1), *ETS1* (Hs00428293_m1), and normalized to *18S* (Hs99999901_s1). For cell lines, Taqman Gene expression assays were used for *EHF*, *TERT* (Hs00972656_m1), *BRAF* (Hs00269944_m1), and *18S*. Samples were run in triplicates on ABI 7900HT Real-Time PCR system (Applied Biosystems) or StepOnePlus Real-Time PCR system (Applied Biosystems). Relative expressions were calculated based on 2^−ΔCt^.

### Western Blot Analyses

Pierce RIPA Buffer (Thermo Fisher Scientific) with 10% Protease Inhibitor Cocktail (Sigma-Aldrich) and 1% phenylmethanesulfonyl fluoride (Sigma-Aldrich) was used for preparation of cell lysates. After quantifications with BCA Protein Assay (Bio-Rad), 30 µg of lysates were separated in NuPAGE 10% Bis-Tris Gels (Invitrogen) and transferred to 0.45 µm nitrocellulose membranes. Membranes were blocked in 5% nonfat milk diluted in Tris-buffered saline/0.5% Tween 20 and incubated with the primary antibodies. The primary antibodies used were: anti-EHF (1:500 dilution, ab167264, Abcam, RRID: AB_3073776), anti-phosphorylated Erk (1:1000 dilution, 9102S, Cell Signaling Technology, RRID: AB_330744), anti-BRAF (1:1000 dilution, sc-5284, Santa Cruz, RRID: AB_626760), anti-GAPDH (1:1,000, 5174, Cell Signaling Technology, RRID: AB_10622025), and anti-Vinculin (1:1000 dilution, sc-73614, Santa Cruz, RRID: AB_1131294). Goat anti-mouse IgG Secondary Antibody (Thermo Fisher Scientific, 62-6520, RRID: AB_2533947) and goat anti-rabbit IgG Secondary Antibody (Thermo Fisher Scientific, 31460, RRID: AB_228341) were used for secondary antibody incubation before detection using SuperSignal West Femto Maximum Sensitivity Substrate (Thermo Fisher). Protein quantification was performed with the ImageJ software using GAPDH or vinculin for normalization purposes.

### Transfections

A customized EHF expression plasmid containing the coding sequence of *EHF* (NM_001206616.2) and corresponding empty control plasmid were purchased from VectorBuilder (Chicago, IL, USA). PTC cell lines (MDA-T32 and MDA-T41) grown in 6-well plates were transfected with 1 µg/well EHF expression or empty control plasmid at 70% to 90% confluence using lipofectamine LTX (Thermo Fisher Scientific) according to the protocol provided by the manufacturer. The customized *BRAF^V600E^* small interfering RNA (siRNA) (target sequence: 5′ UAG CUA CAG AGA AAU CUC G 3′) or siRNA control (Silencer Select Negative Control No. 1 siRNA, Thermo Fisher, 4390843) was transfected into PTC cells with RNAiMAX (Thermo Fisher Scientific) following the manufacturer's protocol. Transfected cells were harvested for subsequent analyses after 48 hours. At least 3 independent biological repeats were performed for each cell line.

### Drug Treatment

Melanoma cell lines (A375 and SK-MEL-3) were treated with 1 μM vemurafenib (Selleck Chem, Houston, TX, USA), a selective *BRAF^V600E^* inhibitor, whereas the control cells were treated with an equal volume of culture medium containing dimethyl sulfoxide (dilution 1:10000, Sigma Aldrich). Following 24-hour treatment, cells were collected for subsequent analysis. The duration and concentration of vemurafenib treatment was according to a previous publication (32).

### Chromatin Immunoprecipitation and qPCR

Chromatin immunoprecipitation followed by quantitative PCR (ChIP-qPCR) was performed to evaluate the direct binding of EHF to the *TERT* promoter region. Briefly, cells from PTC cell lines MDA-T32 and MDA-T41 were cross-linked with 1% formaldehyde for 10 minutes at room temperature to preserve protein-DNA interactions. Cross-linking was quenched with 125 mM glycine for 5 minutes. The cells were then lysed, and the chromatin was sheared to an average fragment size of 200 to 500 bp using micrococcal nuclease (10 U/µL, 15 minutes). The sheared chromatin was immunoprecipitated with 5 μg of antibody specific to EHF (Proteintech, 27195-1-AP, RRID: AB_2880796) or 1 μg normal rabbit IgG antibody (Cell Signaling Technology, 2729, RRID: AB_1031062), at 4 °C overnight with gentle rotation. Protein A/G magnetic beads (Cell Signaling Technology, 9006) were added to capture the antibody-protein-DNA complexes, followed by extensive washing with low-salt and high-salt solutions. The DNA-protein cross-links were reversed by incubation in 65 °C for 4 hours with proteinase K treatment. DNA was then purified using spin columns. Quantitative PCR was performed using SYBR Green with primers specific to the *TERT* promoter region (forward: 5′-CAC CCG TCC TGC CCC TTC ACC TT-3′; reverse: 5′-GGC TTC CCA CGT GCG CAG CAG GA-3′). Enrichment of target sequences was calculated relative to input DNA and normalized to IgG groups.

### Statistical Analyses

Statistical analyses were performed using IBM SPSS Statistics version 25 (IBM, Armonk, NY) and GraphPad Prism 9 (GraphPad Software, San Diego, CA). Mann-Whitney *U* test or Kruskal Wallis test was used for comparison between groups, whereas Spearman rank-order correlation (ρ) was used for correlation analysis of PTC_K_ and PTC_TCGA_ based on the distribution of data. Cox regression and log-rank tests were used for survival analyses and visualized with Kaplan-Meier plots for overall survival (endpoints: dead or alive) and disease-free survival (endpoints: relapse/progression or disease-free). *EHF* mRNA was grouped as low and high expression based on median separation. Paired *t* test was used for comparison between treated and control group in PTC and melanoma cell lines. A *P* value <.05 was considered statistically significant.

## Results

### Differentially Expressed ETS Factors Related to Disease Stage in PTC

**Figure 1.**
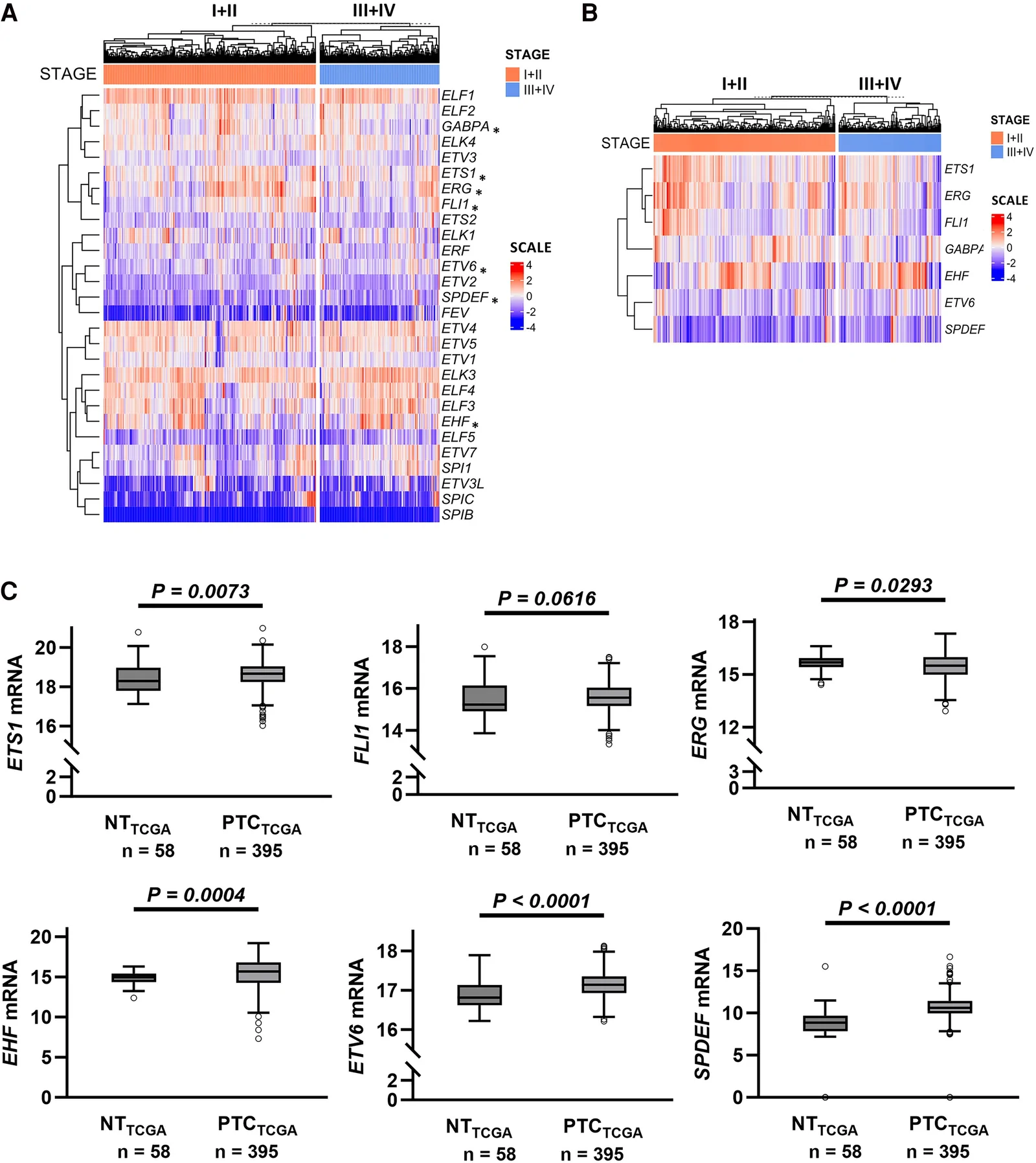
Differentially expressed genes (DEG) analysis of ETS transcription factors in relation to disease stage in PTC and normal thyroid. (A) Heatmap showing the mRNA expression of all analyzed ETS factors. The 28 ETS transcription factors were analyzed in relation to disease stages I + II (n = 252) compared to stage III + IV (n = 142) in PTC_TCGA,_ identifying *GABPA*, *ETS1*, *FLI1*, *SPDEF*, *EHF*, *ERG*, and *ETV6* as differentially expressed between stages with an adjusted *P* value <.05. Statistically significant DEGs are indicated by asterisks (*). (B) Heatmap of the 7 significantly differentially expressed ETS factors. Scale: scaled mRNA expression. (C) Boxplots showing comparison of mRNA expression levels for 6 DEG with differential expression in PTC compared to adjacent nontumorous thyroid tissue (NT_TCGA_).

To identify ETS transcription factors affecting patient outcomes, DEG analysis was performed for the 399 cases in PTC_TCGA_ grouped according to disease stage. Through DEG analysis, 7 of the 28 ETS transcription factors (*ETS1*, *ERG*, *FLI1*, *GABPA*, *EHF*, *ETV6*, and *SPDEF*) were identified as differentially expressed between stage I + II and III + IV with an adjusted *P* value <.05 (Fig. 1A and B and Supplementary Table S3) (29). Among the 7 DEGs, 3 (*SPDEF*, *EHF*, and *ETV6*) showed higher expression in stage III + IV tumors compared to stage I + II, and 4 DEGs (*GABPA*, *ETS1*, *FLI1*, and *ERG*) were expressed at lower levels in stage III + IV compared to stage I + II PTCs. Because *GABPA* was already extensively analyzed in our previous study using the same PTC_TCGA_ and PTC_K_ cohorts, we chose to exclude it from further analyses (21). In that study, we found that lower *GABPA* expression was associated with *TERT* promoter mutation and mRNA expression as well as with several clinicopathological parameters, including tumor size, disease stages, distant metastases, and disease-free survival (21). Comparison between normal thyroid and PTC showed a significant difference for *SPDEF*, *EHF*, *ETV6*, *ETS1*, and *ERG* expressions (Fig. 1C).

### ETS Factors Are Associated With Clinicopathological Features and *BRAF^V600E^* or *TERT* Promoter Mutation in PTC

To further elucidate the relationship between the ETS factors, clinicopathological parameters, and relevant genetic alterations, such as *BRAF^V600E^* and *TERT* promoter mutations, a comprehensive comparison of the differentially expressed ETS factors was performed in PTC_TCGA_ (Table 1, Supplementary Table S4 and Supplementary Figs. S1-4) (29). Here, we found that all 6 differentially expressed ETS factors except for *SPDEF* were associated with at least 1 of the following clinical parameters: age, disease stage, tumor stage, lymph node metastasis, distant metastasis, or extrathyroidal extension. In addition, gene expressions of *EHF*, *ERG*, *FLI1,* and *ETV6* were also associated with *BRAF^V600E^*, whereas *EHF*, *FLI1*, *ETV6*, and *ETS1* were associated with *TERT* expression and/or *TERT* promoter mutations. Given these findings, we proceeded to also analyze the gene expressions of *EHF*, *ERG*, *FLI1*, *ETV6*, and *ETS1* with RT-qPCR in our own cohort PTC_K_ as a validation cohort of the previously mentioned results (Table 2, Supplementary Table S5, and Supplementary Figs. S1-4) (29).

**Table 1.** Comparison of clinical and genetic characteristics to EHF expression in PTC_TCGA_

Parameter (n = informative)	Observations	*EHF* mRNA
**ETS factor mRNA**		(n = 395)
–Median (min-max)		227 (.43-2610)
**Age at diagnosis (n = 399)**		ρ = −.045, *P* = .376
–Median (min-max), y	46 (15-89)	
**Gender (n = 399)**		*P* = .086
–Female/male	n = 290 (73%)/n = 109 (27%)	
**Disease stage (n = 398)**		*P* = .037
–Stage I/II/III/IV	n = 226 (57%)/n = 29 (7%)/n = 94 (24%)/n = 49 (12%)	
**Tumor stage (n = 397)**		*P* = .001
–T1/T2/T3/T4	n = 112 (28%)/n = 126 (32%)/n = 138 (35%)/n = 21 (5%)	
**Lymph node metastasis (n = 373)**		*P* < .001
–Yes/no	n = 209 (56%)/n = 164 (44%)	
**Distant metastasis (n = 252)**		*P* = .245
–Yes/no	n = 4 (2%)/n = 248 (98%)	
**Extrathyroidal extension (n = 382)**		*P* < .001
–None/minimal/moderate or advanced	n = 245 (64%)/n = 119 (31%)/n = 18 (5%)	
***BRAF* V600E (n = 391)**		*P* < .001
–Mutation/wild-type	n = 221 (57%)/n = 170 (43%)	
***TERT* promoter mutation (n = 300)**		*P* = .415
–Mutation (C228T; C250T)/wild-type	n = 31 (10%) (n = 23 ; n = 8)/n = 269 (90%)	
***TERT* mRNA (n = 395)**		*P* = .022
–Yes/No	n = 119 (30%)/n = 276 (70%)	
***TERT* mRNA (n = 395)**		ρ = .113, *P* = .025
–Median (min-max)	0.00 (0.00-103.5)	
**Overall survival (n = 399)**		HR = 1.000, *P* = .428
–Deceased/censored	n = 15 (4%)/n = 384 (96%)	95% CI = .999-1.001
–Follow-up: median (min-max) months	31.4 (0.0-178.2)	
**Disease-free survival (n = 386)**		HR =1.000, *P* = .420
–Relapsed/censored	n = 40 (10%)/n = 346 (90%)	95% CI = 1.000-1.001
–Follow-up: median (min-max) months	30.1 (0.0-178.2)	

Mann-Whitney *U* test or Kruskal Wallis test was used for comparison between groups; Spearman rank-order correlation (ρ) for correlation and univariate Cox regression for survival analyses with gene expressions as continuous variables.

Abbreviation: HR, hazard ratio.

**Table 2.** Comparison of clinical and genetic characteristics to EHF expression in PTC_K_

Parameter (n = informative)	Observations	*EHF* mRNA
**ETS factor mRNA**		(n = 93)
–Median (min-max)		2.183 (0.046-16.022)
**Age at diagnosis (n = 93)**		ρ = .284, *P* = .006
–Median (min-max), y	51 (15-97)	
**Gender (n = 93)**		*P* = .650
–Female/male	n = 67 (72%)/n = 26 (28%)	
**Tumor size (n = 88)**		ρ = .103, *P* = .342
–Median (min-max) cm	2.5 (0.3-12.0)	
**Lymph node metastasis (n = 93)**		*P* = .622
–Yes/no	n = 50 (54%)/n = 43 (46%)	
**Distant metastasis (n = 93)**		*P* = .114
–Yes/no	n = 12 (13%)/n = 81 (87%)	
***BRAF* V600E (n = 93)**		*P* = .010
–Mutation/wild-type	n = 70 (75%)/n = 23 (25%)	
***TERT* promoter mutation (n = 93)**		*P* = .036
–Mutation (C228T; C250T)/wild-type	n = 29 (31%) (n = 24 ; n = 5)/n = 64 (69%)	
***TERT* mRNA (n = 93)**		*P* < .001
–Yes/no	n = 53 (57%)/n = 40 (43%)	
***TERT* mRNA (n = 93)**		ρ = .304, *P* = .003
–Median (min-max)	0.01 (0.00-12.34)	
**Overall survival (n = 93)**		HR = 1.171, *P* = .006
–Dead/alive	n = 32 (34%)/n = 61 (66%)	95% CI = 1.046-1.311
–Follow-up: median (min-max), y	14.8 (0.2-26.5)	
**Disease-free survival (n = 93)**		HR = 1.116, *P* = .105
–Relapsed, progression/no evidence of disease	n = 26 (28%)/n = 67 (72%)	95% CI = .977-1.275
–Follow-up: median (min-max), y	13.5 (0.1-26.5)	

Mann-Whitney *U* test was used for comparison between groups; Spearman rank-order correlation (ρ) was used for correlation and univariate Cox regression was used for survival analyses with gene expressions as continuous variables.

Abbreviation: HR, hazard ratio.

### High *EHF* Expression Is Associated With Poor Clinical Outcomes, *BRAF^V600E^* and *TERT* Expression/Promoter Mutation in PTC

**Figure 2.**
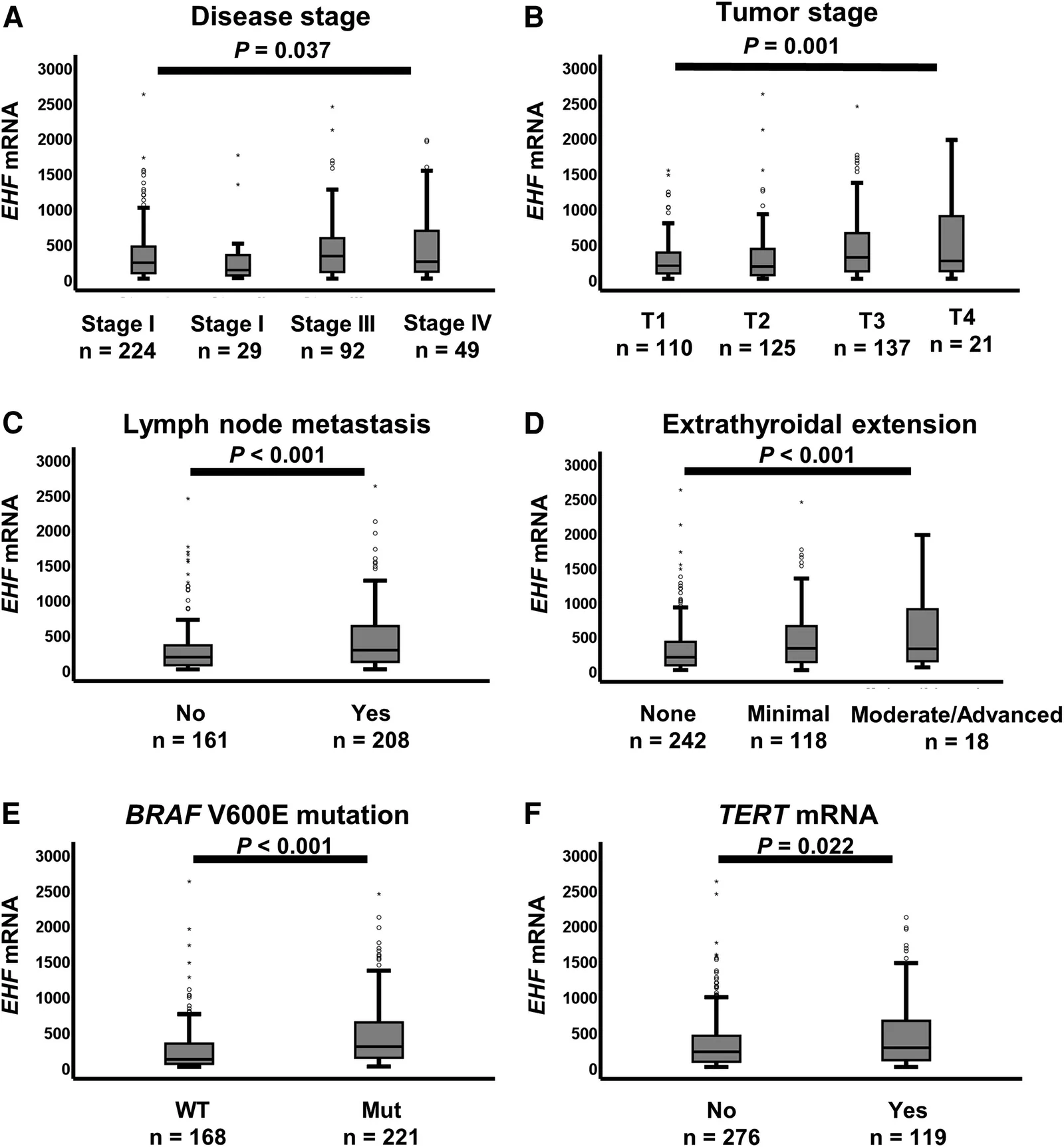
High *EHF* mRNA is associated with poor clinical features, *BRAF^V600E^*, and *TERT* mRNA in PTC_TCGA_. (A-D) High expression of *EHF* is associated with higher stages (A), higher primary tumor stages (B), lymph node metastasis (C), and extrathyroidal extension (D). (E, F) High *EHF* expression is associated with *BRAF^V600E^* mutation (E) and *TERT* mRNA expression (F). Mut, mutant; WT, wild-type.

**Figure 3.**
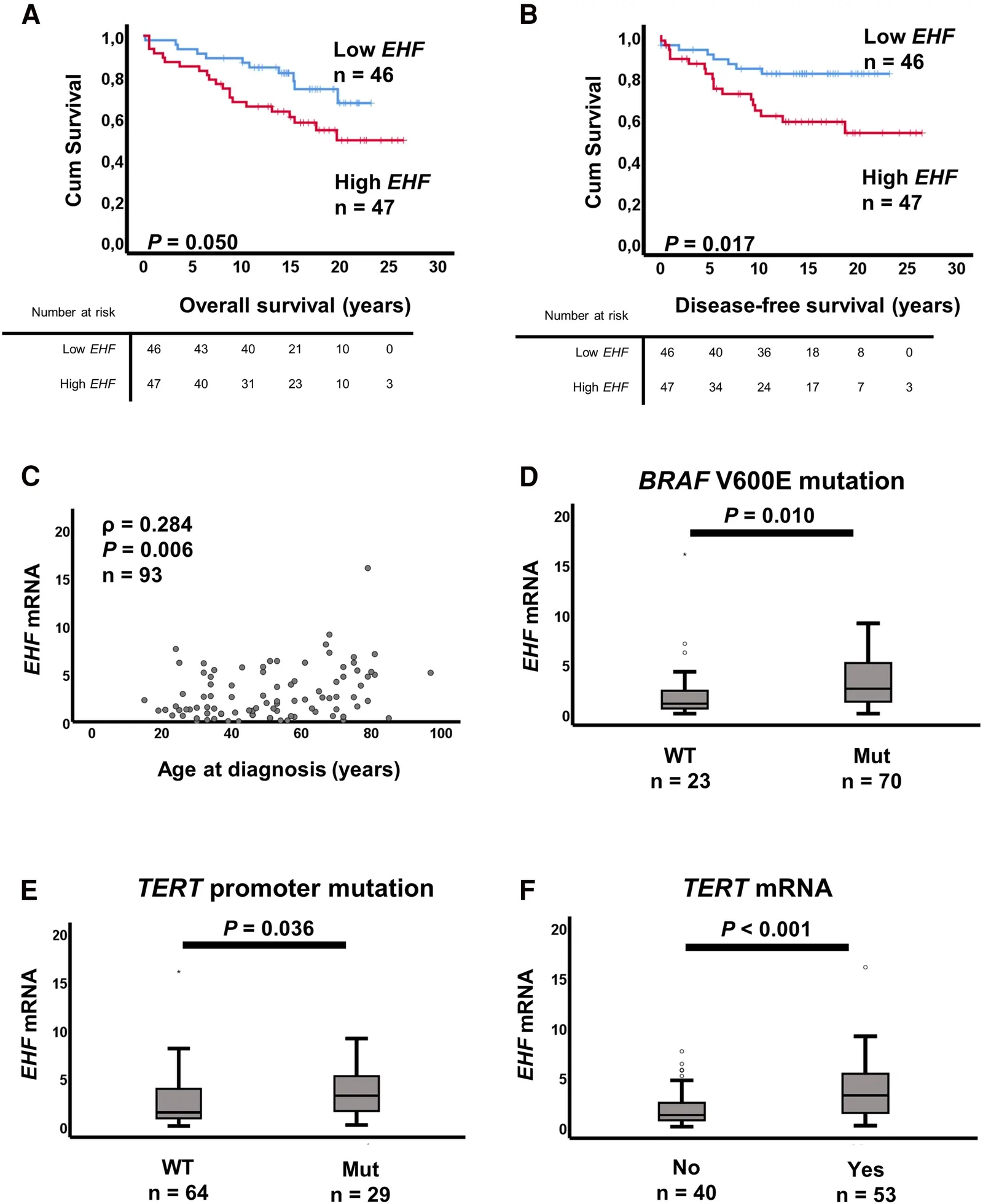
High *EHF* mRNA is associated with poor prognosis, *BRAF^V600E^*, and *TERT* promoter mutation/expression in the PTC_K_ cohort. Relative *EHF* mRNA expression was determined in PTC_K_ using RT-qPCR and analyzed in relation to clinicopathological data and the mutational status of *BRAF^V600E^* and *TERT* promoter. (A, B) Kaplan-Meier survival plots and corresponding numbers at risk presented below. Patients with high *EHF* mRNA exhibited a tendency toward shorter overall survival (A) and significantly shorter disease-free survival (B). (C) *EHF* mRNA levels were positively correlated with older age at diagnosis. (D-F) High *EHF* mRNA was significantly associated with the presence of *BRAF^V600E^* (D), *TERT* promoter mutation (E), and *TERT* expression (F). mut, mutant; WT, wild-type.

After analyzing both PTC_TCGA_ and PTC_K_ cohorts, high expression of *EHF* was found to be consistently associated with aggressive clinical features, *BRAF^V600E^*, and *TERT* promoter mutations/expression (Table 1 and Table 2). Thus, *EHF* emerged as the most promising candidate gene of the significantly differentially expressed ETS factors. In PTC_TCGA_, higher expression of *EHF* was associated with more advanced disease stages (*P* = .037), higher tumor stages (*P* = .001), the presence of lymph node metastasis (*P* < .001), and extrathyroidal extension (*P* < .001) (Table 1 and Fig. 2A-D). As previously mentioned, higher *EHF* expression was also associated with the presence of *BRAF^V600E^* mutation (*P* < .001) and *TERT* expression (*P* = .022), indicating an association between these (Table 1 and Fig. 2E and F). Analysis of PTC_K_ showed that high expression of *EHF* was significantly associated with shorter disease-free survival (*P* = .017); a tendency toward shorter overall survival (*P* = .050) was likewise observed (Fig. 3A and B). Higher *EHF* expression was also positively correlated with older age at diagnosis (ρ = .284, *P* = .006) (Fig. 3C). Consistently, high expression of *EHF* was indeed associated with the presence of *BRAF^V600E^* mutation (*P* = .010), *TERT* promoter mutation (*P* = .036), and *TERT* expression (*P* < .001) in PTC_K_, confirming the findings in PTC_TCGA_ (Fig. 3D-F).

### *EHF* Is Upregulated and Associated With Poor Prognosis in the Presence of *BRAF^V600E^* and *TERT* Promoter Mutation

**Figure 4.**
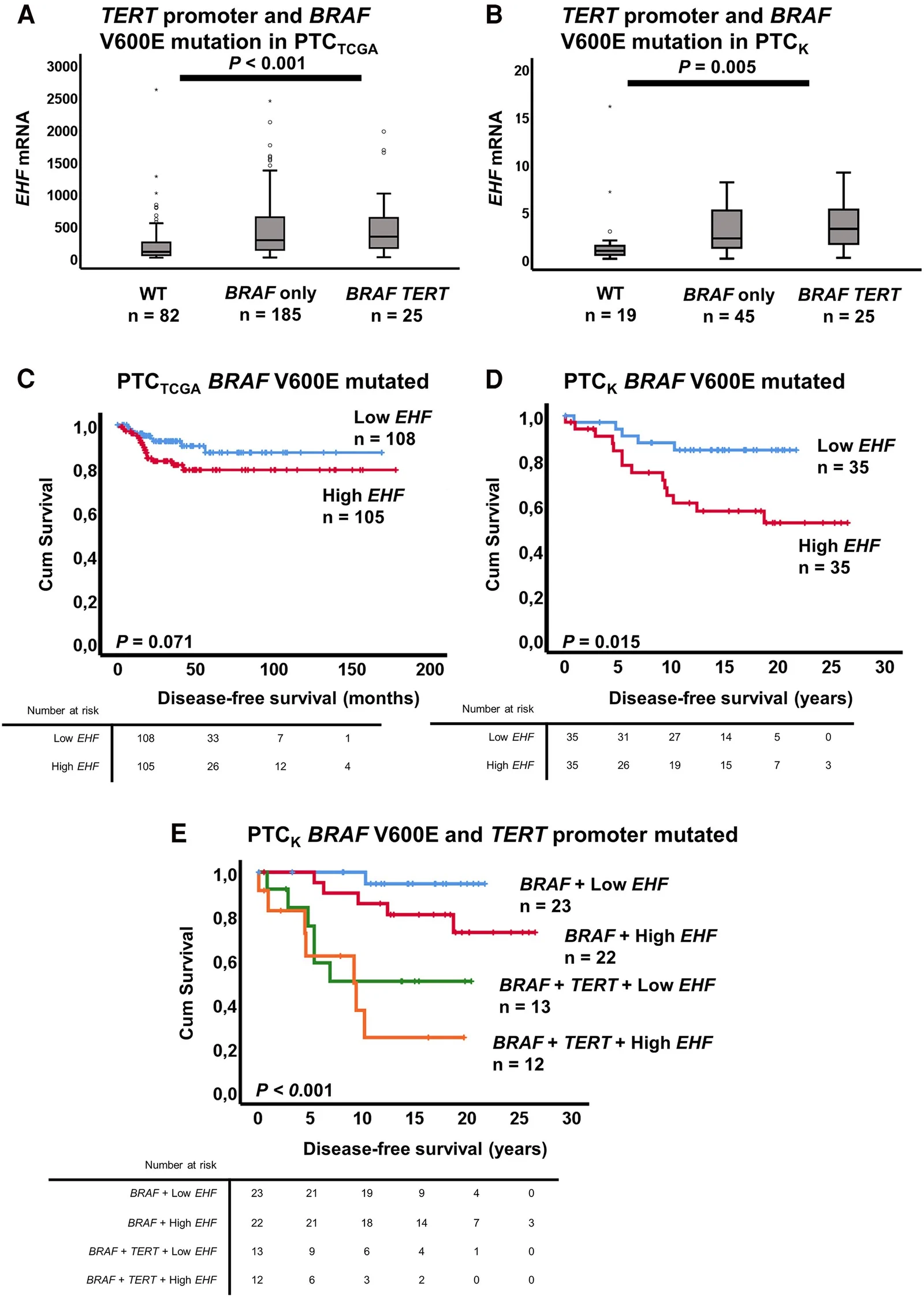
*EHF* expression and its adverse prognostic effect is dependent on *BRAF^V600E^* and *TERT* promoter mutation status. (A, B) Expression of *EHF* was significantly higher in patient groups harboring either *BRAF^V600E^* only or concurrent *BRAF^V600E^* and *TERT* promoter mutation as compared to the double wild-type (WT) group in both PTC_TCGA_ (A) and PTC_K_ (B). (C, D) *BRAF^V600E^* mutated cases with a high level of *EHF* mRNA showed a trend and significant association to shorter disease-free survival in PTC_TCGA_ (C) and PTC_K_ (D), respectively. (E) Patients harboring concurrent *BRAF^V600E^*, *TERT* promoter mutation, and high *EHF* exhibit the shortest disease-free survival in PTC_K_. *EHF* was grouped as high or low based on median separation in each group. BRAF, *BRAF^V600E^*; TERT, *TERT* promoter mutation; WT, wild-type.

Given the hypothesis that the prognostic impact of concurrent *BRAF^V600E^* and *TERT* promoter mutations is mediated by the upregulation of ETS transcription factors, we further explored the relationship of *EHF*, *BRAF^V600E^*, and *TERT*. Interestingly, when comparing the *EHF* expression across groups classified by mutational status of *BRAF^V600E^* and *TERT* promoter mutations, we found that the group with neither *BRAF^V600E^* nor *TERT* promoter mutations had the lowest expression of *EHF* compared to the groups carrying *BRAF^V600E^* only or concurrent *BRAF^V600E^* and *TERT* promoter mutations (Fig. 4A and B). The results were confirmed in both PTC_TCGA_ (*P* < .001) and PTC_K_ (*P* = .005), indicating the upregulation of *EHF* in the presence of *BRAF^V600E^*. Subgroup survival analysis of *BRAF^V600E^* mutated cases also demonstrated an association of high *EHF* expression with shorter disease-free survival in PTC_K_ (*P* = .015); a similar trend was observed in PTC_TCGA_ (*P* = .071) (Fig. 4C and D). However, in subgroup analysis of *BRAF* wild-type cases, no association between high or low *EHF* expression (based on the median level) and disease-free survival was observed in PTC_TCGA_ (*P* = .220) or PTC_K_ (*P* = .463), further indicating a *BRAF*-dependent effect of EHF. We then proceeded by analyzing *BRAF^V600E^* mutated cases while also stratifying for *TERT* promoter mutations and identified that the group harboring concurrent mutations along with high *EHF* expression exhibited the shortest time to relapse or progression in PTC_K_ (*P* < .001) (Fig. 4E) but not in PTC_TCGA_ (data not shown). Similar findings were observed using a Cox regression model in PTC_K_, showing that the group harboring both mutations and high *EHF* was significantly associated with shorter disease-free survival compared to the mutation negative group with low *EHF* expression (Table 3).

**Table 3.** Cox regression of BRAF^V600E^, TERT promoter mutation, and EHF mRNA in PTCK (n = 89)

		Disease-free survival				
	Observations	B coefficient	HR	SE	95% CI	*P* value
						.006
WT + low *EHF*	n = 10 (11%)					
WT + high *EHF*	n = 9 (10%)	1.091	2.978	1.227	0.269-32.979	.374
*BRAF* + low *EHF*	n = 23 (26%)	−0.800	0.449	1.414	0.028-7.188	.572
*BRAF* + high *EHF*	n = 22 (25%)	0.789	2.200	1.097	0.256-18.899	.472
*BRAF* + *TERT* + low *EHF*	n = 13 (15%)	1.882	6.570	1.082	0.789-54.724	.082
*BRAF* + *TERT* + high *EHF*	n = 12 (13%)	2.392	10.935	1.072	1.336-89.478	.026

*EHF* mRNA is grouped as low/high based on median separation in each group.

Abbreviations: *BRAF*, *BRAF^V600E^*; HR, hazard ratio; *TERT*, *TERT* promoter mutation; WT, wild-type for *BRAF^V600E^* and *TERT* promoter mutation.

### EHF Increases *TERT* Expression in the Presence of *BRAF^V600E^* and *TERT* Promoter Mutation and Is Downregulated by *BRAF^V600E^* Inhibition

In summary, our results from PTC_TCGA_ and PTC_K_ indicate that the adverse clinical effect of *EHF* could be mediated by acting as a mechanistic link between *BRAF^V600E^* and *TERT* promoter mutations (Supplementary Fig. S5A), which was further studied in vitro (29). For this purpose, we first determined the basic *EHF* and *TERT* expressions and validated the *BRAF^V600E^* and *TERT* promoter mutational status in TC cell lines (Supplementary Fig. S5B and C) (29).

**Figure 5.**
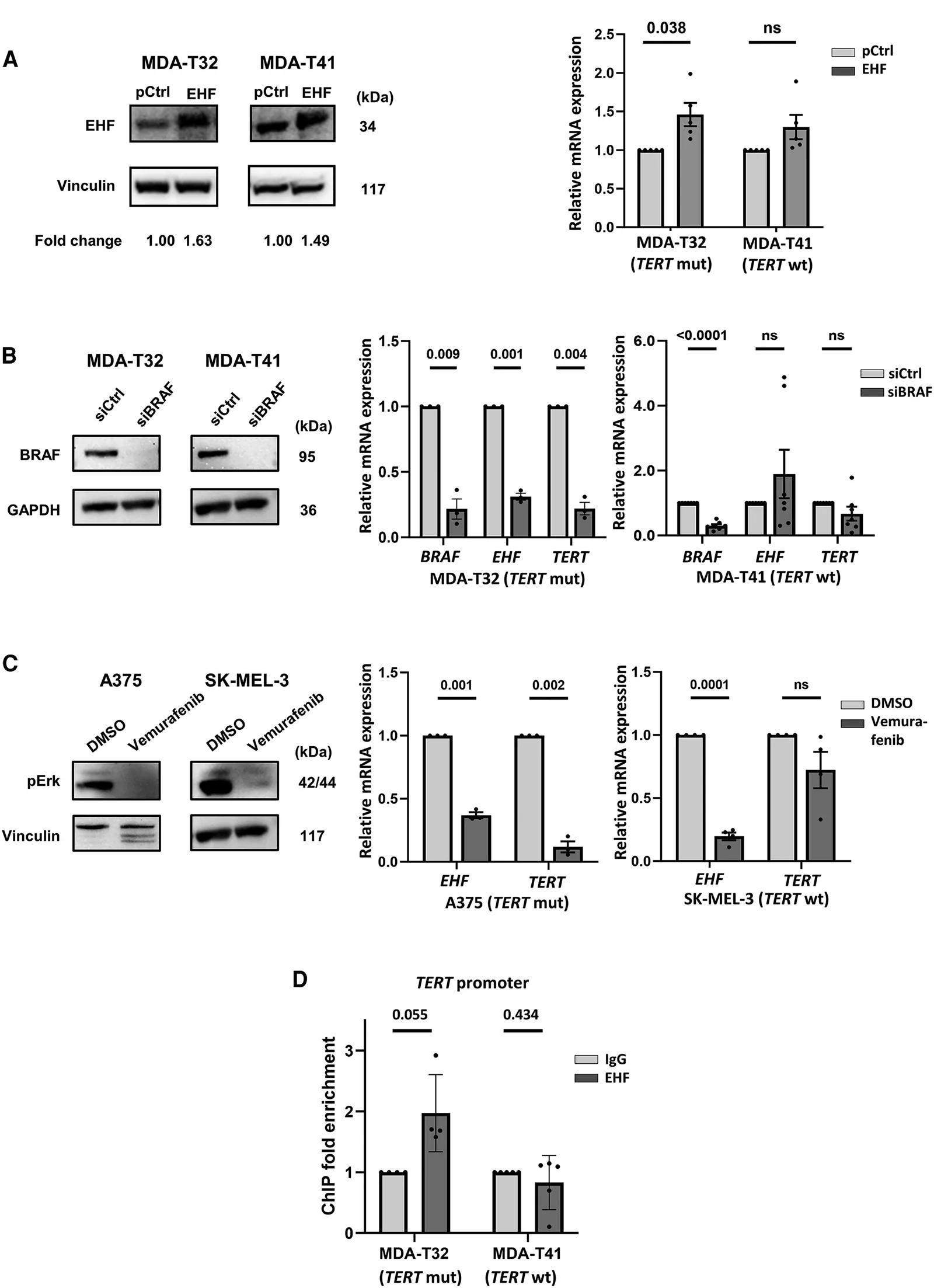
EHF increases *TERT* expression in cells harboring concurrent *BRAF^V600E^* and *TERT* promoter mutation and is decreased on *BRAF^V600E^* inhibition. (A) PTC cells were transfected with EHF expression vector (EHF) and empty control vector (pCtrl) and harvested after 48 hours for analysis of *TERT* mRNA. Overexpression of EHF significantly increased *TERT* mRNA in PTC cells harboring concurrent *BRAF^V600E^* and *TERT* promoter mutation (MDA-T32), but no significant (ns) difference was observed in PTC cells with *BRAF^V600E^* only (MDA-T41). The transfection efficiency was confirmed with Western blot analysis in both cell lines. (B) PTC cell lines were transfected with *BRAF^V600E^* siRNA (siBRAF) and control siRNA (siCtrl) and were collected after 24 hours for analysis of *BRAF, EHF*, and *TERT* mRNA using RT-qPCR. Knockdown of *BRAF^V600E^* significantly decreased *EHF* and *TERT* mRNA in cells harboring concurrent *BRAF^V600E^* and *TERT* promoter mutations, but not in the cells with *BRAF^V600E^* only. The efficiency of siRNA knockdown was confirmed with Western blot and RT-qPCR. (C) Melanoma cell lines were treated with 1 μM vemurafenib for 24 hours followed by *EHF* and *TERT* mRNA quantifications using RT-qPCR. The efficiency of *BRAF^V600E^* inhibition was confirmed with decreased phosphorylated Erk (pErk) expression through Western blotting. Pharmacological inhibition of *BRAF^V600E^* significantly decreased *EHF* mRNA in both cell lines. However, a significant decrease of *TERT* mRNA expression was only observed in melanoma cells harboring concurrent *BRAF^V600E^* and *TERT* promoter mutation (A375), but not in the cells with *BRAF^V600E^* only (SK-MEL-3). (D) The direct binding of EHF to the *TERT* promoter region was assessed in PTC cell lines MDA-T32 and MDA-T41, using chromatin immunoprecipitation followed by qPCR (ChIP-qPCR). The bar graphs show the relative enrichment of the *TERT* promoter region after immunoprecipitation with an anti-EHF antibody compared to a nonspecific IgG control. EHF binding to the *TERT* promoter exhibited a trend towards increased enrichment in *TERT* promoter mutant MDA-T32 cells, although the difference did not reach statistical significance. No enrichment of EHF binding was observed in *TERT* promoter wild-type MDA-T41 cells.

To explore if EHF affects *TERT* expression, we transfected the *BRAF^V600E^* and *TERT* promoter mutated MDA-T32 cells and the *BRAF^V600E^* only MDA-T41 cells with an EHF-expressing plasmid. The efficacy of transfection was confirmed with Western blotting in both cell lines. Indeed, our results demonstrated that *TERT* expression significantly increased when overexpressing EHF in PTC cells harboring concurrent *BRAF^V600E^* and *TERT* promoter mutation (*P* = .038) but not in PTC cells harboring *BRAF^V600E^* only (Fig. 5A).

To further investigate the potential effects of *BRAF^V600E^* on *EHF* and *TERT* expressions, customized *BRAF^V600E^* siRNA were transfected into PTC cell lines (MDA-T32 and MDA-T41) to knock down the *BRAF^V600E^* expression (Fig. 5B). The efficiency of transfection was confirmed with Western blotting and RT-qPCR. Gene expression analyses demonstrated a significant decrease of *EHF* and *TERT* expressions upon *BRAF^V600E^* knockdown in PTC cells with concurrent *BRAF^V600E^* and *TERT* promoter mutation but not in cells with a *BRAF^V600E^* mutation only (Fig. 5B).

In addition, melanoma cells lines (A375 and SK-MEL-3) were treated with vemurafenib to inhibit the *BRAF^V600E^* induced MAPK-activation. The efficiency of vemurafenib treatment was confirmed with Western blotting using phosphorylated Erk as a downstream target control. Pharmacological inhibition of *BRAF^V600E^* in A375 cells with concurrent *BRAF^V600E^* and *TERT* promoter mutations showed a significant decrease in both *EHF* and *TERT* expressions. However, in SK-MEL-3 cells that harbor *BRAF^V600E^* only, a significant decrease was only observed for *EHF* but not *TERT* expression (Fig. 5C). Taken together, our results indicate that *EHF* could be upregulated by *BRAF^V600E^* and in turn possibly upregulate *TERT* expression in the presence of a *TERT* promoter mutation.

Finally, the direct binding of EHF to the *TERT* promoter was evaluated using ChIP-qPCR in both *TERT* promoter mutant (MDA-T32) and wild-type (MDA-T41) PTC cell lines (Fig. 5D). In the *TERT* promoter mutant cells, a fold enrichment of 1.97 (mean value) in EHF was observed, although the difference did not reach a statistical significance (*P* = .055). However, no enrichment in EHF was shown in the *TERT* promoter wild-type cells. These findings suggest a potential binding of EHF to the mutated *TERT* promoter but not to the wild-type *TERT* promoter.

## Discussion

In this study, we explored the role of ETS transcription factors in relation to clinical phenotype in PTC. The ETS transcription factors is one of the largest evolutionary well-conserved transcription factor families and plays an important role in tumorigenesis (17). So far, 28 ETS genes have been identified in human and they function by regulating genes involved in important biological processes including signaling cascades, proliferation, differentiation, apoptosis, migration, invasion, and angiogenesis (33). Altered ETS factor expression and function is hence strongly associated with tumor progression in various types of cancer, including TC (17, 22-25, 33). In our analysis, *GABPA*, *ETS1*, *FLI1*, and *ERG* were expressed at lower levels in stage III + IV compared to stage I + II and *SPDEF*, *EHF*, and *ETV6* showed higher expression in stage III + IV tumors compared to stage I + II. Except for *SPDEF*, all of these genes were associated with at least 1 clinical parameter, thus indicating a clinically relevant, predictive role of the ETS factors in PTCs. It is known that the expression of ETS factors and their functions are influenced by the cellular context and that both upregulation and downregulation can contribute to tumorigenesis through either activation of oncogenic or loss of tumor suppressive activities, respectively (17).

We then proceeded to validate these findings in a local cohort of PTCs, and the results from both cohorts combined identified *EHF* as the most promising candidate gene. Even though the 2 clinical cohorts differ from each other in terms of clinical variables as well as the method used for mRNA expression analyses, high *EHF* expression was consistently associated with adverse clinical features, *BRAF^V600E^* and *TERT* promoter mutation/expression. However, it should be emphasized that we selectively analyzed PTCs with a predominant nonfollicular growth pattern to confidently exclude NIFTP lesions from both the TCGA and Karolinska cohorts. Consequently, it remains uncertain whether our results are applicable to infiltrative follicular variant PTCs, which represent a *BRAF*-driven entity characterized by a predominant follicular growth pattern.

EHF has been studied in various types of cancer including gastric (34), prostate (35), pancreatic (36), and ovarian cancer (37), with evidence of both oncogenic and tumor-suppressive functions. Previously, it was reported that *EHF* is upregulated in PTC compared to nontumorous thyroid tissue (38) and our analysis confirmed these results. EHF knockdown in TC cell lines also inhibited proliferation, colony formation, invasion, and migration, whereas EHF overexpression exhibited the opposite effect (38). In addition, inhibition of EHF repressed tumor growth in nude mice in the same study, providing both in vitro and in vivo evidence of the oncogenic effects of EHF in TC (38).

Given previous reports suggesting a mechanistic link between *BRAF^V600E^*, ETS factors, and *TERT* promoter mutation in PTC (16), we investigated if the adverse clinical effect of *EHF* could be related to this hypothesis. We found indeed that *EHF* expression is strongly associated with *BRAF^V600E^* and *TERT* promoter mutation/expression in our analyzed cohorts. These observations are consistent with reports from a previous transcriptomic study where *EHF* was identified as upregulated in the patient groups harboring *BRAF^V600E^* only or concurrent *BRAF^V600E^* and *TERT* promoter mutations compared to the patient group without mutations (16). In our own cohort, survival analyses also demonstrated that the group harboring both mutations along with high *EHF* exhibited the shortest time to relapse/progression, suggesting that the adverse clinical effect of *EHF* could well be mediated by this mechanism. Further, ectopic expression of EHF increased *TERT* expression in PTC cell lines harboring concurrent *BRAF^V600E^* and *TERT* promoter mutations, supporting the potential link between *EHF* and *TERT*. Moreover, subsequent interventions with pharmacological inhibition or siRNA knockdown of *BRAF^V600E^* resulted in decreased *EHF* and *TERT* expressions in cell lines harboring concurrent *BRAF^V600E^* and *TERT* promoter mutations. However, in MDA-T41, which harbors *BRAF^V600E^* only, no significant alteration in *EHF* expression was observed on siRNA inhibition as would be expected. Interestingly, MDA-T41 also harbor *RB1* and *TP53* mutations where loss of *RB1* has previously been reported as disrupting BRAF signaling in *BRAF^V600E^*-mutated melanomas (39, 40).

The direct binding of several ETS factors, including GABPA, ETS1, and ETS2, to the mutant *TERT* promoter has been demonstrated in various cancers (41-43). However, no report has been published regarding the binding of EHF to the *TERT* promoter. To evaluate the direct binding of EHF to the mutant *TERT* promoter region in PTC, ChIP-qPCR assay was conducted in our study. To our knowledge, this study is the first to report the potential binding of EHF to the mutated *TERT* promoter in PTC.

Vemurafenib is a targeted therapy for melanomas that harbor the *BRAF^V600E^* mutation, selectively inhibiting Erk phosphorylation in *BRAF* mutant cells (44). Notably, our investigation in PTC cell lines MDA-T32 and MDA-T41 failed to demonstrate a decrease in phosphorylated Erk expression after vemurafenib treatment (data not shown), in contrast to the favorable response observed in some other TC cell lines (45). This may result from the adaptable transition between the RAF isoforms, where *ARAF* has been reported as activated by BRAF inhibitors, which consequently stabilize the MAPK pathway leading to BRAF inhibitor resistance (46, 47). In addition, the activation of *HER2* and *HER3*, which interestingly has been identified as targets of EHF, might also contribute to the resistance to vemurafenib in *BRAF*-mutated thyroid cancer cells (38, 48). The inconsistence of vemurafenib treatment efficiency across different TC cell lines corresponds to the critical understanding of underlying resistance mechanisms for further potential therapeutic approaches in PTC (49).

Targeting ETS factors could be an attractive therapeutic option in cancers (17). For instance, YK-4-279 has demonstrated efficacy in inhibiting ETS across various cancers, such as thyroid, lymphoma, prostate, neuroblastoma, and melanoma (50-54). Additionally, the small molecule BRD32048 has shown efficiency in inhibiting the cancer cell invasion and proliferation in melanoma and prostate cancer cells by targeting ETS (55). Although ETS factors primarily function as transcriptional regulators, their effects may extend beyond the regulation of specific genes alone. Hence, it is crucial to explore the comprehensive regulatory roles of ETS factors before therapeutic applications can be realized. Also, the potential synergistic interactions of ETS factors inhibitors and BRAF inhibitors to prevent drug resistance has to be further evaluated.

In this study, some limitations may be considered when interpreting the results. There are differences between the 2 clinical cohorts PTC_TCGA_ and PTC_K_ and the methods used for gene expression analyses. Moreover, the regulation of *BRAF^V600E^* by vemurafenib was demonstrated in melanoma cell lines, but not the 2 PTC cell lines in our study because of their resistance. Additionally, the *TERT* promoter hotspot mutation C228T was evaluated functionally by ChIP-qPCR, but not the C250T mutation.

In summary, we have identified several potentially clinically relevant members of the ETS transcription factor family in PTC, where *EHF* was indicated as the most promising candidate. Concurrent *BRAF^V600E^* and *TERT* promoter mutation with upregulated *EHF* are identified in PTC patients with the most aggressive clinical course. Moreover, in vitro experiments suggest a regulatory effect of *BRAF^V600E^* on *EHF* expression, which further potentially regulates mutant *TERT* promoter leading to increased *TERT* expression.

## Data Availability

Original data generated and analyzed during this study are included in this published article or in the data repositories listed in References.

## Abbreviations

ATC: anaplastic thyroid carcinoma
ChIP: chromatin immunoprecipitation
DEG: differentially expressed gene
NIFTP: neoplasm with papillary-like nuclear feature
PTC: papillary thyroid carcinoma
RT-qPCR: reverse transcriptase quantitative PCR
siRNA: small interfering RNA
TC: thyroid carcinoma
TCGA: The Cancer Genome Atlas
WHO: World Health Organization
