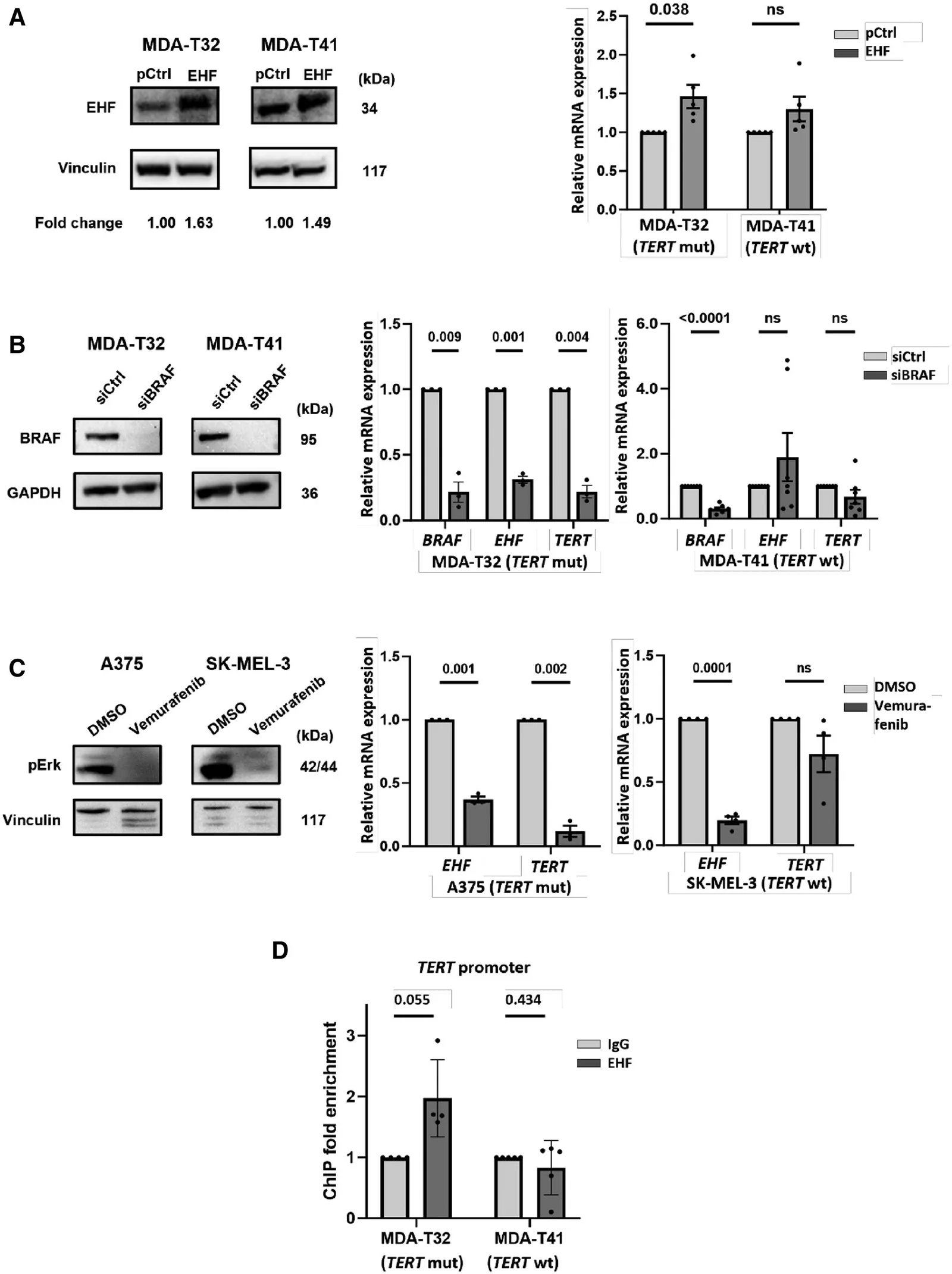# Correction to: “BRAF-induced *EHF* Expression Affects TERT in Aggressive Papillary Thyroid Cancer”

**DOI:** 10.1210/clinem/dgag353

**Published:** 2026-09-01

In the above-named article by Xu Y, Gao J, Wang N, Zedenius J, Nilsson I-L, Lui W-O, Xu D, Juhlin CC, Larsson C, Mu N, (*J Clin Endo Metab*. 2025; 110(3): 693-705; doi: 10.1210/clinem/dgae589), there was an error in Figure 5C.

In the originally published article, in Figure 5, Figure 5C included an incorrect vinculin panel for the SK-MEL-3 cell line due to an inadvertent figure assembly error.

Figure 5C has been corrected below with the appropriate vinculin lane from the original experiment. The corrected figure accurately reflects the original experimental data, and this correction does not alter the results, interpretation or conclusion of the study.

The authors regret the error.

doi: 10.1210/clinem/dgae589

**Original Figure 5**

**Figure:**
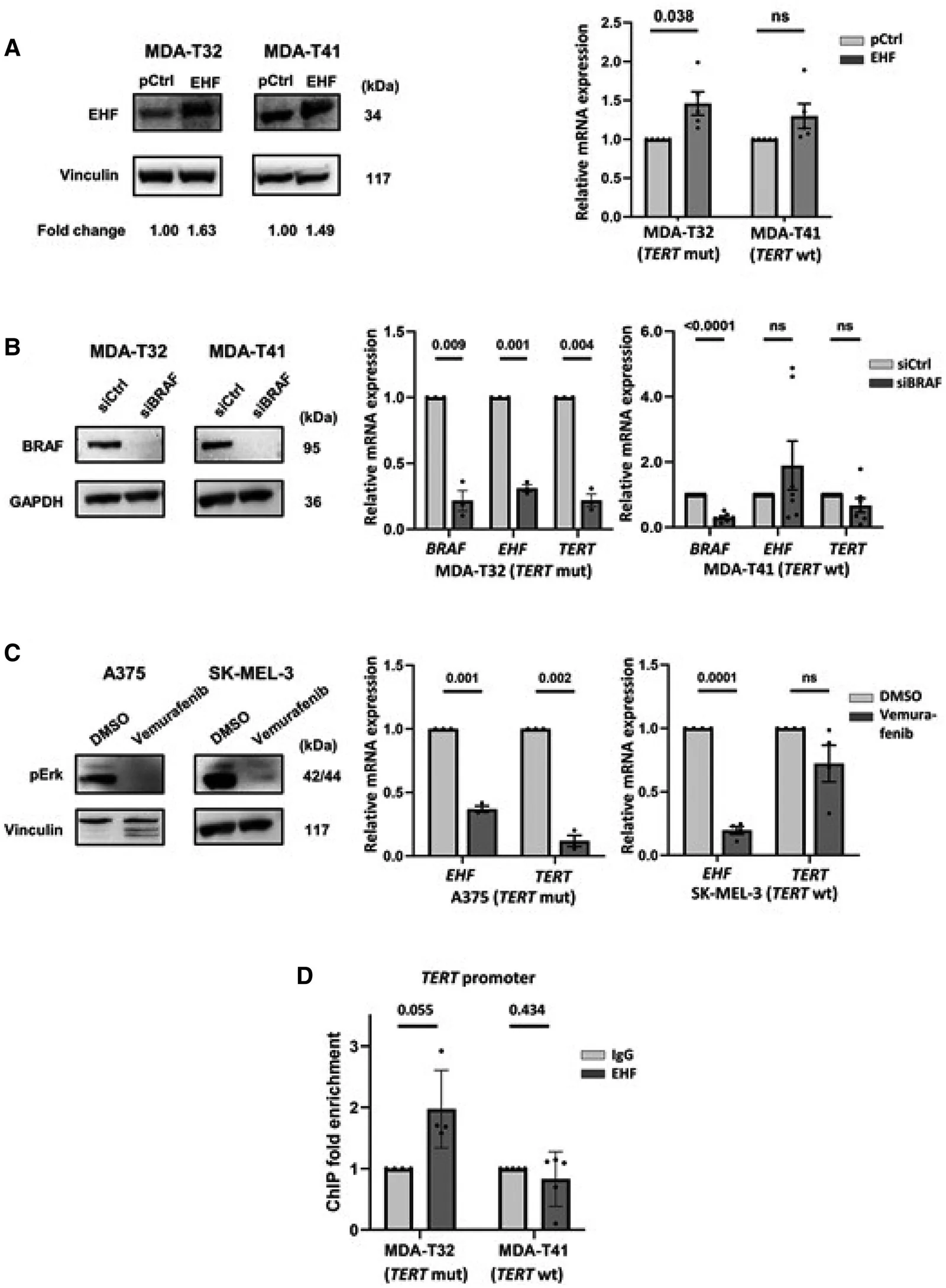


**Corrected Figure 5:**

**Figure:**